# Efficacy and Low Toxicity of Normo-Fractionated Re-Irradiation with Combined Chemotherapy for Recurrent Glioblastoma—An Analysis of Treatment Response and Failure

**DOI:** 10.3390/cancers16213652

**Published:** 2024-10-29

**Authors:** Niklas Benedikt Pepper, Nicholas Grischa Prange, Fabian Martin Troschel, Kai Kröger, Michael Oertel, Tanja Kuhlmann, Michael Müther, Oliver Grauer, Walter Stummer, Hans Theodor Eich

**Affiliations:** 1 Department of Radiation Oncology, University Hospital Muenster, 48149 Muenster, Germany; 2 Department of Neuropathology, University Hospital Muenster, 48149 Muenster, Germany; 3 Department of Neurosurgery, University Hospital Muenster, 48149 Muenster, Germany; 4 Department of Neurology with Institute of Translation Neurology, University Hospital Muenster, 48149 Muenster, Germany

**Keywords:** re-resection, radiotherapy, patterns of relapse, radiochemotherapy, high-grade glioma

## Abstract

Background: Glioblastoma is the most common malignant brain tumor in adults. Even after maximal safe resection and adjuvant chemoradiotherapy, patients normally relapse after a few years or even months. Standard treatment for recurrent glioblastoma is not yet defined, with re-resection, re-irradiation, and systemic therapy playing key roles. Usually, re-irradiation is combined with concurrent chemotherapy, harnessing the radiosensitizing effects of alkylating agents. Methods: A retrospective analysis of 101 patients with recurrent glioblastoma treated with re-irradiation was conducted, evaluating the survival impact of concurrent chemotherapy regimens, as well as prior resection. Patients were subcategorized according to concurrent chemotherapy (temozolomide vs. CCNU vs. combination of both vs. none) and details are given regarding treatment toxicity and patterns of relapse after first- and second-line treatment. Results: Patients were treated with normo-fractionated re-irradiation (with prescription dose of ~40 Gy to the PTV), resulting in a moderate cumulative EQD2 (~100 Gy). The mean overall survival was 11.3 months (33.5 months from initial diagnosis) and mean progression free survival was 9.5 months. Prior resection resulted in increased survival (*p* < 0.001), especially when gross total resection was achieved. Patients who received concurrent chemotherapy had significantly longer survival vs. no chemotherapy (*p* < 0.01), with the combination of CCNU and TMZ achieving the best results. Overall survival was significantly better in patients who received the CCNU + TMZ combination at any time during treatment (first or second line) vs. monotherapy only. The treatment of larger volumes (mean PTV size = 112.7 cm^3^) was safe and did not result in worse prognosis or increased demand for corticosteroids. Overall, the incidence of high-grade toxicity or sequential radionecrosis (5%) was reasonably low and treatment was tolerated well. While second-line chemotherapy did not seem to influence patterns of relapse, patients who received TMZ + CCNU as first-line treatment had a tendency towards better local control with more out-field recurrence. Conclusions: Normo-fractionated re-irradiation appears to be safe and is accompanied by good survival outcomes, even when applied to larger treatment volumes. Patients amenable to undergo re-resection and achieving concurrent systemic therapy with alkylating agents had better OS, especially when gross total resection was possible. Based on existing data and experiences reflected in this analysis, we advocate for a multimodal approach to recurrent glioblastoma with maximal safe re-resection and adjuvant second chemoradiation. The combination of TMZ and CCNU for patients with methylated MGMT promoter yielded the best results in the primary and recurrent situation (together with re-RT). Normo-fractionated RT enables the use of more generous margins and is tolerated well.

## Simple Summary

In this single-center retrospective analysis of 101 patients with recurrent glioblastoma, we demonstrate the benefit of a multimodal treatment approach with normo-fractionated re-RT with combined alkylating chemotherapy after surgical resection. Additionally, questions of patient selection, treatment tolerability, and patterns of relapse are addressed. Multimodal Treatment with re-resection followed by re-irradiation in combination with alkylating chemotherapy achieved the best results, especially when a combination of two agents (TMZ and CCNU) is used. Treatment was tolerated well with little evidence of radionecrosis or hematological toxicity and mean overall survival was 11.3 months (mean progression free survival: 9.5 months). No influence of second-line chemotherapy on patterns of relapse was detected.

## 1. Introduction

Glioblastoma (GBM) is the most frequent malignant brain tumor in adults. Originating from cerebral glioma cells, this malignancy confers a dismal prognosis: Even after optimal treatment, progression-free survival (PFS) is generally limited to 1–2 years [1]. The fast recurrence or progression of tumor growth is oftentimes associated with limited overall survival and neurologic decline, leading to impaired quality of life (QoL) [2,3].

For initial treatment, maximal safe resection is a key pillar of therapy. The use of fluorescence-guided resection has proven beneficial regarding the extent of tumor resection, leading to increased rates of gross total resection (GTR) [4]. Surgical resection is followed by adjuvant chemoradiotherapy (CRT): 60 Gy in 30 fractions is applied and prescribed to a clinical target volume (CTV), defined as the surgical cavity with the inclusion of surrounding contrast-enhancing lesions with an additional margin of 15 mm, also including sites of potential invasion based on pre-treatment MRI [5]. Therefore, high doses to large intracerebral volumes are frequent. Based on the paradigm-shifting EORTC-NINC trial, the 6-week-course of radiotherapy (RT) is usually combined with concomitant and sequential oral temozolomide (TMZ) [6,7]. In the NOA9/CeTeG-trial, a dual chemotherapy combining TMZ with CCNU resulted in improved overall survival (OS) compared to TMZ alone in patients with methylated O-6-methylguanine-DNA methyltransferase (MGMT) promoter [6,8]; it has gained popularity as a concomitant chemotherapy for first-line treatment [9]. Additionally, the application of alternating tumor treating fields (TTFs) during sequential chemotherapy should also be discussed as a treatment option, based on increased survival rates demonstrated in the EF-14 trial [10].

Nevertheless, relapse after several months is the common course of the disease. With tumor recurrence or progression, therapy options are limited, and no clear standard is defined [6]. Relapse management typically involves assessment of re-resection, re-irradiation (re-RT), and/or chemotherapy. Different strategies for re-RT have been explored, using normo-fractionated or hypo-fractionated regimes (with 25–45 Gy in 5–22 fractions), resulting in a moderate cumulative EQD2 (~100 Gy), or even stereotactic radiosurgery (using single-fraction treatment with doses between 10 and 20 Gy) [1]. Since local recurrence at the primary site is the most common pattern of relapse [11], a second course of RT commonly requires a direct overlap of treatment fields with sites of initial irradiation, inherently limiting dose decisions by normal tissue tolerance. While the recent randomized controlled RTOG1205 trial [12] has reinforced the role of re-irradiation for patients with recurrent glioma, many questions, like optimal prescription dose and fractioning, target volume definition, and the role of accompanying systemic therapy, remain unanswered.

This study describes a large cohort treated with or without concurrent alkylating chemotherapy during normo-fractionated re-irradiation and aims to draw conclusions regarding patient selection, target definition, and choice of concurrent chemotherapy.

## 2. Materials and Methods

a. Patient selection

We retrospectively reviewed all cases of patients with cerebral re-irradiation of glioblastoma treated in the radiation oncology department of our tertiary care cancer center between January 2015 and December 2022. Records were screened for treatment of initial and recurrent disease, including applied radiation schedules and concurrent chemotherapy, as well as clinical and radiologic follow-up data after re-irradiation. Data reporting follows the STROBE-guidelines for reporting observational studies.

b. Chemoradiotherapy

**Figure 1:**
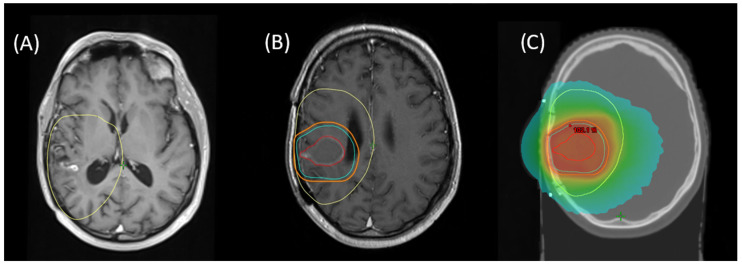
Example for (**A**) MRI of recurrent blood–brain barrier disruption of the right temporal lobe as a manifestation of recurrent glioblastoma, fused with initial RT planning CT to show first-line treatment PTV (yellow). (**B**) Planning MRI after surgical resection for fusion with planning CT with resulting target volumes for re-RT (GTV = red, CTV = cyan, PTV = orange). (**C**) Resulting cumulative dose distribution for re-irradiation with 39.6 Gy via helical therapy.

Re-RT planning was performed in accordance with ICRU8325. All patients were treated with 1.8–2 Gy daily fractions 5/week with IMRT-planning for conformal treatment volumes. All treatment plans and target volume definitions of patients included in this cohort were assessed. Treatment planning for all patients was realized via Varian Eclipse (by Varian medical systems, Palo Alto, CA, USA) or TomoTherapy^®^ (by Accuray Inc., Sunnyvale, CA, USA). Diagnostic MRI was co-registered with the planning CT for all patients. Patients were treated with immobilization by a thermoplastic mask, using 6 MV or 15 MV Photons in different beam setups (Intensity Modulated Radiation Therapy [IMRT] or Volumetric Intensity Modulated Arc Therapy [VMAT], with helical therapy as a special form of VMAT). Re-RT target volumes were defined as standardized by ICRU50 with the gross tumor volume (GTV) enclosing the primary disease site (defined as sites of recurrent or progressing tumor after initial treatment, marked by blood–brain barrier disruptions in contrast-enhanced T1- and T2-MRI), the clinical target volume (CTV) incorporating sites of suspected subclinical infiltration (also considering the T2-FLAIR-signal if clinically appropriate), and the planning target volume (PTV) creating an additional margin to compensate for possible incongruences in patient positioning (usually 3–5 mm). For patients who received prior re-resection, the CTV was defined as areas of supposed residual tumor or associated with a high risk of subclinical infiltration, based on postoperative MRI (T2 and contrast-enhanced T1) with an additional margin, based on disease location and size, prior treatment, extent of resection, and clinical performance status. Postoperative patients with delayed initiation of radiation treatment (>3 weeks) received a dedicated planning MRI. An example for target volume definition and dose distribution is given in Figure 1.

Concomitant chemotherapy was administered based on histopathological specifications, prior treatment, and disease dynamic after discussion with the multidisciplinary neuro-oncological tumor board and initiated with the beginning of re-RT.

c. Treatment toxicity and follow-up

For all patients, regular follow-ups were conducted quarter-annually. All records of follow-up visits in the departments of radiation oncology, neurosurgery, and neuro-oncology were evaluated for signs of treatment toxicity in the form of neurological symptoms or increased intracranial pressure in need of pharmacological treatment (i.e., corticosteroids, reported as equivalent dose of dexamethasone), as well as laboratory signs of systemic treatment toxicity in the form of the depletion of blood work parameters or increased hepatic enzymes in the regular laboratory examinations during and post-treatment.

As part of follow-up, patients had regular quarter-annual appointments in the departments of neurosurgery, neuro-oncology, and radiation oncology, where performance status, signs of treatment toxicity, and subsequent MRI imaging were reviewed and remission status was critically reassessed. Participation in at least one follow-up program was deemed mandatory for inclusion in this analysis. Suspected disease progression was discussed in the tumor board. For this analysis, reports of patient’s follow-up visits and radiological reports were reviewed for an overall clinical development and performance score, as well as disease progression/relapse or radiological changes as possible manifestations of treatment toxicity, such as radiation necrosis. As part of this clinical routine, patient cases with suspected tumor progression were discussed in the interdisciplinary neurooncological tumor board and the MRI was critically reviewed. In cases of unclear response assessment, FET-PET/MRI imaging was initiated, with potential biopsy to confirm suspected progression, if necessary. For this analysis, the available MRIs of patients with confirmed progression (following RANO [response assessment in neuro-oncology] criteria [13]) was co-registered to the treatment planning imaging to analyze patterns of relapse, following the method described by Buglione et al. [14]: “in-field-recurrence” was defined as >80% of recurrent tumor covered by initial PTV, “marginal recurrence” was defined as 80–20% of recurrent tumor covered by initial PTV, and “out-of-field” recurrence was defined as <20% of recurrent tumor covered by initial PTV.

Statistical analysis of survival parameters and influencing factors was conducted using GraphPad Prism 10 (Version 10.1.2 by GraphPad Software, Inc., Boston, MA, USA), using Spearman’s rank test for the correlation of independent variables, as well as log-rank or the Wilcoxon test for survival analysis in a univariate setting and multiple linear regression and the Cox proportional hazard ratio for multivariate modeling.

## 3. Results

a. Patients and demographics

Overall, 115 patients were identified, of whom 101 were eligible for analysis. Fourteen patients were excluded due to insufficient data regarding initial diagnosis and/or treatment or not participating in follow-ups (n = 7), spinal manifestations of GBM (n = 2), not fitting the definition of re-irradiation type A or B [15] (n = 2), or not completing re-RT (n = 3). The mean initial progression-free survival (PFS) between the end of first-line RT treatment and beginning of re-RT was 19.4 months (median: 12 months). For all patients, treatment regimens were determined after discussion with the interdisciplinary tumor board of our certified neuro-oncological cancer center. Most patients received re-resection of the tumor prior to re-RT (n = 62, 61.4%). For those patients, the postoperative MRI was evaluated for suspected residual tumor. All patients with remaining contrast-enhancing tumor sites in the postoperative MRI were categorized as subtotal resection (STR, n = 40, 64.5% of resections) as opposed to gross total resection (GTR, n = 22, 35.5% of resections). A total of 15 patients received biopsy only, and for 24 patients, re-RT was initiated without histological verification in cases where surgery or biopsy was not recommended or refused by the patient. Ten patients were initially diagnosed with Isocitrate Dehydrogenase-1 (IDH-1)-mutant glioblastoma and would now be considered IDH-mutated astrocytoma WHO-grade 4. As they were characterized and treated as GBM at the time of treatment, they were still included in the analysis. Characteristics regarding demographics, initial treatment, performance status, and treatment prior to re-irradiation of the included 101 patients are summarized in Table 1.

b. Radiotherapy, systemic therapy, and monitoring

The prescription dose to the PTV ranged from 19.8 to 45 Gy. In total, 14 patients received an additional boost (ranging from 5.4 to 39.6 Gy) to enhance the RT dose in previously uninvolved areas (i.e., outside of the initial PTV), or to increase the dose to the GTV if PTV coverage with doses of 39.6 Gy or higher was limited due to adjacent organs being at risk (e.g., brainstem, chiasm, or optic nerves).

**Figure 2:**
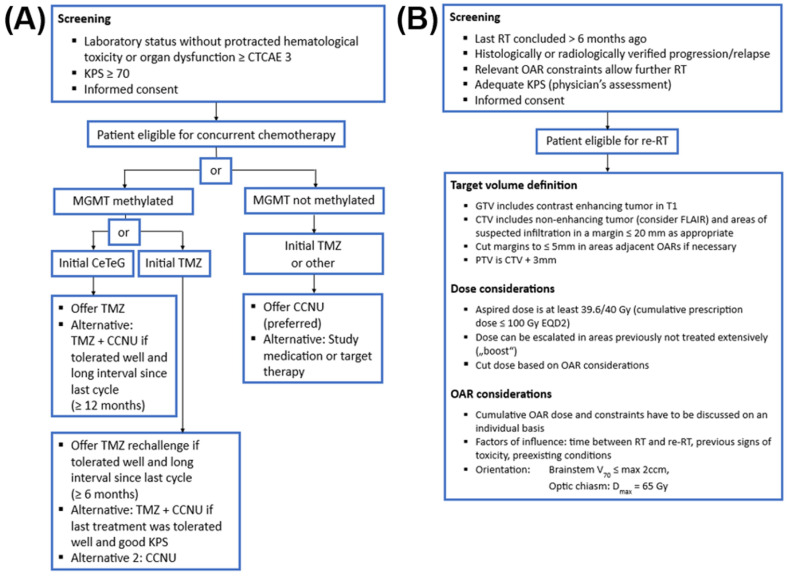
Management approach for decision-making concerning (**A**) re-RT and (**B**) concurrent systemic therapy. Abbreviations: RT: radiotherapy; KPS: Karnofsky performance status; GTV: gross tumor volume; CTV: clinical target volume; PTV: planning target volume; EQD2: equivalent uniform dose of 2 Gy; V70: volume receiving 70 Gy; Dmax: maximal dose; CTCAE: Common Terminology Criteria of Adverse Events; MGMT: O-6-methylguanin-DNA-methyltransferase; TMZ: temozolomide; CCNU: lomustine; CeTeG: combined chemotherapy with TMZ and CCNU.

All patients received concomitant systemic treatment during first-line treatment in the form of TMZ (n = 86), a combination of TMZ and CCNU (n = 11), or the study treatment with systemic therapy (n = 4, all patients received TMZ + Nivolumab or placebo (blinded) as part of the Checkmate 548 trial). A total of 80 patients (79.2%) received concomitant oral chemotherapy in the form of either CCNU (110 mg/m^2^ d1 in cycles of 42 days), Temozolomide (75 mg/m^2^ daily during re-RT, followed by cyclic application with 150–200 mg/m^2^ d1–5 in cycles of 28 days), or a combination of both in accordance with the NOA09/CeTeG protocol (CCNU 100 mg/m^2^ d1 + TMZ 100 mg/m^2^ d2–6 in cycles of 42 days). An approach to decision-making regarding re-CRT prescription is illustrated in Figure 2. All patients received bloodwork and clinical examinations for signs of treatment toxicity at least on a weekly basis but with intensified frequency if necessary. In case of neurological symptoms like dizziness, headache, or vertigo, oral corticosteroids were prescribed with caution and reduced as soon as possible, based on symptom development. GTV and PTV size details, dose ranges and details on overlap with initial treatment, and the necessity and dynamic of steroid intake (comparing start and end of re-irradiation) are given in Table 2. Table 3 summarizes the details concerning chemotherapy schedules and laboratory signs of toxicity (according to CTCAE Vers. 5). Most patients had concluded initial chemotherapy (91%) at the point of recurrence. Of note, two patients received TMZ-rechallenge chemotherapy before concluding the first six cycles but both did not suffer hematological toxicities > grade 1. An additional five patients received TMZ-rechallenge <6 months after the conclusion of the last cycle; one of them developed grade 3 hepatic toxicity (increased AST). One patient in the TMZ + CCNU cohort had an early progression 3 months after the conclusion of initial radiochemotherapy with TMZ and received combination systemic therapy + re-irradiation, with maximal hematological toxicity of grade 2 thrombopenia. Further specifics (including MGMT methylation status) of the different chemotherapy groups are described in Table A1 in the Appendix A.

c. Follow-Up and survival analysis

At the time of analysis (17 months after the last included patient completed re-RT), 93 patients died (92%). For the surviving patients, OS was calculated to the last registered follow-up visit. The mean overall survival (OS) from the beginning of re-RT was 11.4 months (95%-CI: 9.2–13.5, median: 8 months) and 33.5 months from initial diagnosis (95%-CI: 28.9–38.2, median: 24 months). Imaging leading to the diagnosis of further progression was available for 57 patients; 13 patients showed radiological signs of radiation necrosis in MRI and received additional PET-MRI. Necrosis was confirmed in five cases (via FET-PET/MRI with two patients receiving additional biopsy as PET imaging was not conclusive). The mean PFS in patients was 8.8 months (95%-CI: 5.5–11.4, median: 6 months).

The overall survival rates (OSRs) from the beginning of re-RT were as follows: 6-month OSR: 71%, 9-month OSR: 47%, and 1-year OSR: 37%.

**Figure 3:**
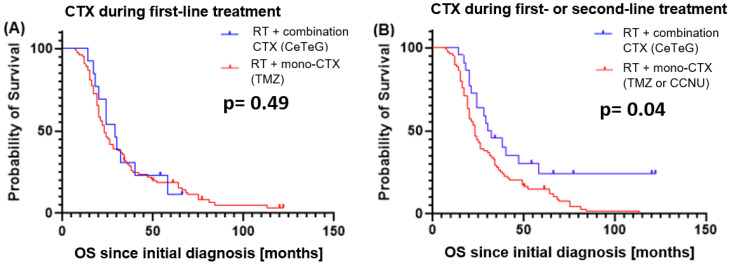
Overall survival comparison in months from initial diagnosis when (**A**) comparing whether or not patients received combination chemotherapy with TMZ and CCNU (CeTeG) during first-line treatment (vs. TMZ), and (**B**) comparing whether or not patients ever received combination chemotherapy with TMZ and CCNU (CeTeG) during the course of first- or second-line treatment (vs. monotherapy). Abbreviations: CTX: chemotherapy; TMZ: temozolomide; CCNU: lomustine; CeTeG: TMZ + CCNU.

When comparing initial treatment with TMZ or CeTeG, no significant difference was observed in PFS (median 11 vs. 17 months, *p* = 0.19) and OS (median 24 vs. 29 months, *p* = 0.49). However, OS was significantly better in patients who received the CCNU + TMZ combination at any time during treatment (first or second line) vs. monotherapy only (median 31 vs. 24 months, *p* = 0.04, HR 0.53). This difference remained significant if patients who did not receive chemotherapy in second-line treatment were excluded from the analysis (*p* = 0.028, HR 0.56). Corresponding Kaplan–Meier-curves are depicted in Figure 3.

**Figure 4:**
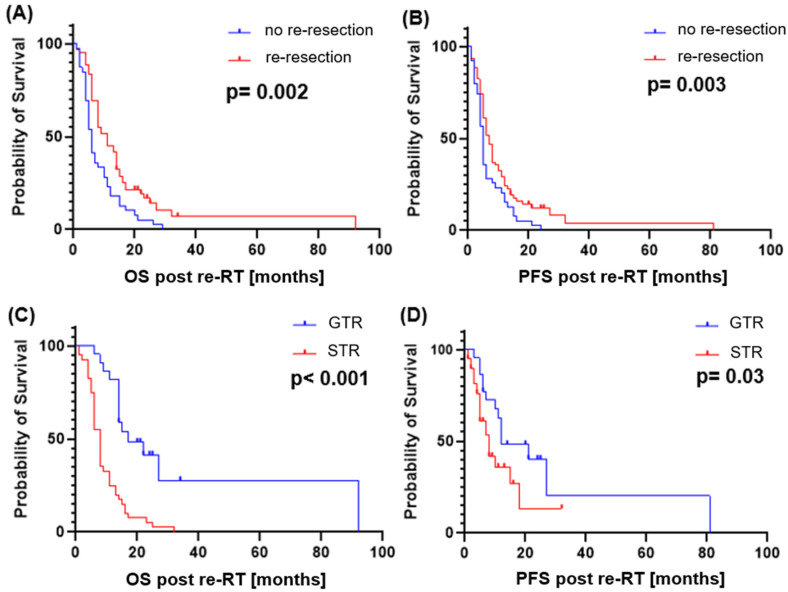
Survival Analysis in dependence of surgical intervention and resection status: (**A**) overall survival (OS) and (**B**) progression-free survival (PFS) in patients with or without re-resection prior to re-RT, as well as the OS (**C**) and PFS (**D**) difference after gross total resection (GTR) as opposed to subtotal resection (STR).

Statistical analysis showed a significant impact of re-resection on survival: a second surgery performed vs. biopsy/no intervention resulted in longer OS (median 11 vs. 6 months, *p* = 0.002, HR: 0.45) and PFS (*p* = 0.03, HR: 0.58). GTR was associated with significant longer survival compared to STR in resected patients (median OS 17 vs. 8 months, *p* < 0.001, HR 0.3). Figure 4 shows the corresponding Kaplan–Meier-curves.

For further analysis, patients were categorized according to chemotherapy administered concomitant to re-RT. The distribution of age and gender, initial therapy, and PFS, as well as MGMT methylation status and other prognostic factors according to two prognostic scores (RRRS [16] and DKTK-ROG [17]) are detailed in Table A1 in the Appendix A.

**Figure 5:**
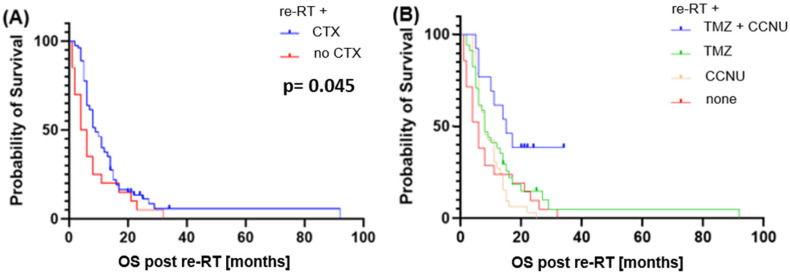
Kaplan–Meier curve of overall survival (**A**) comparing no concomitant chemotherapy vs, chemotherapy (regardless of regimen) and (**B**) depicting different regimens of chemotherapy. Abbreviations: TMZ: temozolomide; CCNU: lomustine.

Detailed analysis of survival showed a significant increase in OS when systemic therapy was applied concomitantly to re-RT (median OS 9 vs. 5 months, *p* = 0.045, HR: 0.55). Comparing different regimes, OS was increased for patients receiving CCNU when compared to no chemotherapy (median OS 8 vs. 5 months *p* = 0.027, HR: 0.86), with comparable results when using TMZ (median OS 8 vs. 5 months, *p* = 0.03, HR: 0.63). No significant difference was found when comparing concomitant CCNU and TMZ (*p* = 0.76). Using combined TMZ and CCNU in second-line treatment showed significant better OS (median OS 15 months) when compared to CCNU (*p* = 0.002, HR: 0.35) and TMZ (*p* = 0.05 HR: 0.48). Differences for PFS were not significant. Corresponding Kaplan–Meier curves are depicted in Figure 5.

Treatment toxicity analysis showed higher rates of hematological adverse events for patients treated with combination chemotherapy, especially higher rates of leukopenia and higher grade (>grade 2) thrombocytopenia when compared to CCNU and TMZ. The incidence of radiation necrosis after re-RT was split evenly between the cohorts (1/1/2/1). No statistically significant correlation was detectable between increased demand for corticosteroid intake and PTV size (*p* = 0.18), increased GTV-to-PTV margins (*p* = 0.52) or applied re-RT dose (*p* = 0.26). A detailed listing of side effect ratios during different types of chemotherapy can be found in Table A2 in the Appendix A.

When comparing actual OS with predicted OS using prognostic scores, the predictive validity was better for DKTK-ROG prognostic score (median overall deviation from predicted survival: 0.5 months, survival longer than predicted in 52 cases, shorter in 49 cases) than for the RRRS (median overall deviation from predicted survival: 2.2 months; survival longer than predicted in 65 cases, shorter in 36 cases). Comparing the different cohorts, the TMZ + CCNU cohort outperformed the predicted survival the most (median additional survival: 8.5 months more than DKTK-ROG prediction; 10/13 patients lived longer than predicted), while survival was overestimated for patients who did not receive chemotherapy (median −2.1 months, 13/21 patients lived shorter than predicted).

**Figure 6:**
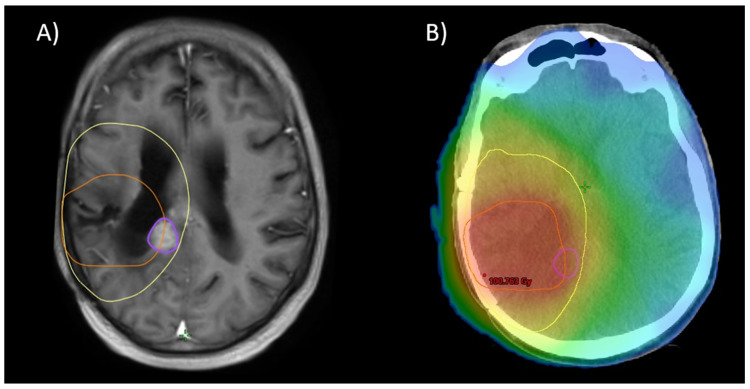
As depicted in Figure 1, 12 months after re-RT: (**A**) Further progression (purple) adjacent to the initial treatment site. Notice the site of progression was covered by the initial PTV (yellow) and partially covered by the re-RT PTV (orange). (**B**) shows a color wash dose distribution of a plan sum of initial RT and re-RT.

For 57 patients, the MRIs of further recurrence were all available in digital form. The patterns of relapse in these patients are described in Table 4, and a visual example is given in Figure 6. This analysis reveals no difference between the subgroups with in-field and marginal recurrence as the majority of cases (28 in-field recurrences, 26 marginal, 3 out-field). However, analysis of the initial patterns of relapse (leading to re-RT) reveals a tendency towards more out-of-field recurrence for patients treated with CeTeG (30% out-field vs. 14% for patients treated with TMZ, *p* = 0.11).

A univariate analysis of patient parameters also revealed statistically significant increased OS in patients with the following characteristics: time between initial RT and re-RT > 12 months (*p* < 0.001), age ≤ 50 years (*p* = 0.01), and initial ECOG < 2 (*p* = 0.002). Parameters that did not show significant impact on OS were re-RT prescription dose > 40 Gy (*p* = 0.62), GTV-to-PTV-margin size (*p* = 0.6), or PTV size > 47 mL (*p* = 0.16), sex (*p* = 0.07), MGMT-status (0.18), or increased demand on corticosteroids during treatment (*p* = 0.35). When analyzing overall survival from initial diagnosis, MGMT promoter methylation was highly significantly linked to prolonged survival (*p* < 0.001, HR 0.45, mean OS 31 vs. 20 months).

Multivariate modeling revealed significant influence of age ≤ 50 years (HR: 1.89 *p* ≤ 0.05), time between initial RT and re-RT > 12 months (HR: 2.21 *p* ≤ 0.05), and resection status (HR: 0.64 *p* ≤ 0.05) while no significance was obtained for ECOG and the application of concurrent systemic therapy. Since MGMT status was only of significant predictive value in the analysis of OS after initial diagnosis, but not when analyzing OS after re-RT, it was not included in the modeling.

## 4. Discussion

This study investigates a large cohort of 101 patients treated with re-irradiation of recurrent GBM over the course of 7 years. With assessment of treatment response and failure as well as influencing factors of survival parameters, this retrospective analysis offers additional information to help answer remaining questions regarding the benefit of multimodal second-course treatment.

Key aspects of our findings are the following:

1. A trimodal approach to recurrent GBM with re-resection, re-RT, and concurrent chemotherapy offers favorable survival rates.
2. Normo-fractionated re-irradiation is safe, revealing low rates of radionecrosis, even in cases of large target volumes.
3. Concurrent systemic therapy is tolerated well and improves survival.
4. A combination of CCNU and temozolomide seems to improve outcomes compared to single-agent therapy.
5. Patterns of relapse indicate no impact of second-line therapy regarding location of recurrence but show a trend towards less in-field progression after first-line treatment for patients receiving TMZ + CCNU.

Recently, the randomized prospective phase II RTOG1205 trial demonstrated that the addition of a second course of radiotherapy to the application of bevacicumab (BEV) increases PFS for patients with recurrent GBM [12]. This was the first randomized prospective trial to demonstrate a survival benefit of re-RT in this disease. For patients with high grade glioma, therapeutic options are limited considering extensive first-line treatment. Anti-VEGF therapy in the form of BEV has been approved by the FDA in this context, based on the demonstrated PFS benefits and reduction in RT side effects [18], but remains mostly unavailable in Europe where no approval has been granted. With a clear second-line treatment missing and inclusion in clinical trials not always manageable for patients, the treatment options to be considered remain the same as for initial treatment: re-evaluation of surgery, radiotherapy, and/or systemic therapy [6,19]. Yet, poor prognosis and limitations due to prior treatment demand for careful evaluation of the risk-to-benefit ratio on an individual basis.

While experiences in treatment tolerability and effectiveness have grown over the last decades, and, consequently, cerebral re-RT has become a more common approach among radiation oncologists, several key aspects remain unanswered:

1. What target volume should be treated and what dose is appropriate?

While clear guidelines regarding dose prescription and target volume definition exist for primary glioblastoma treatment, re-irradiation with extensive treatment fields and high doses is mostly limited due to exhausted dose constraints. Unfortunately, disease recurrence is commonly manifested as local relapse [11], leaving a demand for dose escalation in pretreated areas. In our cohort, the majority of cases were also classified as in-field (70% of available data) or marginal recurrence (14%). No consensus exists regarding the exceedance of the standard QUANTEC dose constraints in this situation, but mixed cohort publications hint towards higher-than-expected tolerability of organs at risk, rendering cerebral second- or even third-course RT possible under careful evaluation of individual risk–benefit ratios [20]. In order not to increase the risk of side effects (with potentially devastating effects on QoL), dose and target volume size need to be compromised regularly. Decisions regarding prescription dose oftentimes start with the question of fractionation, i.e., normo-fractionated or hypo-fractionated RT [1]: while the latter might offer shorter treatment time and suspected biological advantages against radioresistant cells, hypofractionation is mostly reserved to smaller target volumes [1]. Thus, this form of RT mostly focusses on treating contrast-enhancing tumor sites with reaching high EQD2-doses. Stereotactic radiosurgery has also been explored for GBM-recurrence as a means to apply high-dose radiotherapy to small target volumes, also achieving similar results [1,21,22]. On the other hand, normo-fractionated RT can be safely applied to larger volumes with little risk of radionecrosis, but treatment courses take longer [1]. Our analysis demonstrates the safety and efficacy of normo-fractionated re-RT to large target volumes (mean PTV size = 112.7 cm^3^) with inclusion of not only contrast enhancing tumor but also areas of suspected infiltration to a reasonable degree into re-irradiation treatment volumes (in analogy to first-line treatment). Our strategy to escalate doses in areas previously not irradiated to the full extent of 60 Gy during initial treatment unfortunately did not translate to better survival in patients where this was possible, leaving the question unanswered if dose escalation in re-RT is beneficial.

FET-PET-imaging might also offer a way to aid in target definition and potentially enable focused treatment to high-risk areas, reducing the risk of radiation necrosis [23]. Two upcoming prospective randomized trials of the German neurooncological working group (NOA) evaluate the benefits of different fractionation concepts [24] and biologically driven target volume definitions (PET vs. MRI) [25] for recurrent glioma. The RTOG1205-trial has already proven hypo-fractionated re-RT to be safe for small treatment volumes [12] (only including recurrent tumors ≤ 6 cm with an optional CTV expansion of 5 mm for tumors ≤ 3.5 cm) with little side effects when accompanied by BEV (which is also the treatment of choice to the main side effect of high dose re-RT, radionecrosis [18]). In our cohort, the dose and target volume definition were based on contrast-enhancing tumors but with additional margins including sites of suspected subclinical spread, as commonly practiced in first-line therapy (including non-enhancing tumor based on T2/FLAIR if clinical appropriate, resulting in margins of up to 20 mm) and treatment was not limited to small tumors (mean GTV size 25 cm^3^). Nevertheless, treatment tolerability was good. An increase in demand for corticosteroids was seen in 32% of patients, but while other publications attest negative prognostic value regarding survival parameters to the need of steroid treatment [26], this was not the case in our cohort (*p* = 0.35). Furthermore, higher demand for corticosteroids did not correlate significantly with applied dose (*p* = 0.26), PTV size (*p* = 0.18), or margin size (*p* = 0.52) and only five patients developed radionecrosis (in line with expected rates [1]). This underlines that treatment of larger fields is not only safe but causes manageable side effects and areas of non-enhancing tumor can be included if clinically feasible. However, treatment with larger GTV-to-CTV margins did not show a significant impact on PFS (*p* = 0.43) or OS (*p* = 0.6). Regarding the dose concept of normo-fractionated re-RT, our analysis not only shows adequate tolerability but also good outcomes, as survival parameters not only outperformed predicted outcomes based on prognostic scores (DKTK-ROG and RRRS), but also had better progression-free and overall survival than in the RTOG1205 trials combined modality arm and reported values are near the upper limit of reported survival rates in a recent Cochrane meta-analysis of treatment of recurrent glioma [19].

Future research approaches are expected to explore the relevance of volumetric variations in target volumes during treatment for adaptive radiotherapy via MRI linac, which offers new possibilities to individualize and adapt RT planning, as well as potentially deliver higher doses safely and more accurately [27]. This process might be aided by increasing our knowledge of radiomics to identify relevant targets and discriminate tumor growth and treatment-related tissue reactions as well [28]. The clinical impact of this technology remains to be determined.

2. Do patients benefit from multimodal treatment?

Aggressive tumors generally demand multimodal treatment to increase the effectiveness of treatment. With prognosis severely limited, prolonging survival has to be carefully weighed against preserving QoL. This is a challenge that is especially fierce for surgical approaches since the side effects of extensive resection can be severe. Nevertheless, our cohort clearly demonstrates the survival benefit of re-resection, as have previous trials [29,30,31,32]. The OS benefit for patients with GTR was also statistically increased (*p* < 0.001), and its value underlined by remaining a significant factor in our multivariate analysis, but this is most certainly only achievable in selected cases. For patients not suitable for re-resection, another NOA trial evaluates the use of photodynamic therapy (PDT), a method of minimally invasive local tumor treatment [33,34].

The combination of alkylating chemotherapy to re-RT alone also increases survival [35], which was also demonstrated in our analysis (*p* = 0.045). Based on its frequent use in the control arms of several trials, CCNU is oftentimes used for second-line treatment [36] but did not show preferable results in our cohort when compared to TMZ (*p* = 0.18), which also is known as a valid option, especially in MGMT-methylated tumors [37]. A combination of TMZ and CCNU, as proposed for first-line treatment by the NOA09/CeTeG trial, showed the best results in our analysis with significantly increased OS compared to single-agent regimens, but also increased rates of thrombopenia. Interestingly, while no increased OS was found for patients receiving combination chemotherapy as first-line vs. TMZ mono (in contrast to results of the NOA09/CeTeG-trial), a significant difference was found when comparing patients who had received combination CTX vs. never (median 31 vs. 24 months, *p* = 0.04, HR 0.53). In this regard, the absence of an OS benefit for patients receiving the CeTeG regime during first-line treatment should cautiously be interpreted considering the fact that some patients were treated with TMZ + CCNU as a second-line treatment after first-line TMZ monotherapy. Our analysis of survival from the initial diagnosis clearly demonstrates the benefit of using combination chemotherapy (*p* = 0.04), suggesting that the combination of two alkylating agents per se is beneficial and should not be limited to first-line treatment.

Other options for simultaneous treatment have also been investigated in the context of re-RT: While bevacicumab has demonstrated positive results in several retrospective and prospective trials [12,38,39], it remains without approval in Europe. The multi-kinase inhibitor regorafenib has also been evaluated as a treatment option [40], but while the phase II REGOMA trial showed promising results [41], they could not be repeated in the GBM AGILE trial [42,43]. Since regorafenib has also been linked to increased toxicity [44], it was not applied in our cohort as the role of this multi-kinase inhibitor remains to be determined. In this regard, future results of the N2M2 umbrella trial on the combination of target therapy and re-RT are expected to raise interesting implications regarding further individualization of treatment, based on tumor biology [45].

Contemporary efforts to further increase treatment effectiveness also include early and extended inclusion of TTfields [46] and/or radiosensitizers [47,48,49] in the treatment algorithm. Increasing local control, leading to more distant relapse patterns [50], also increases reserves for re-RT, enabling the safer application of higher treatment doses without sacrificing OAR dose constraints. Additionally, a longer time between first- and second-line treatment also was predictive for longer OS after re-RT in our multivariable Cox regression; therefore, aggressive treatment in the first line should be pursued.

Nevertheless, multimodal and combination therapy show increased potential for side effects, which is why careful screening for treatment eligibility is necessary.

3. Who should be considered for re-treatment?

Several working groups have tried to better define the patient collective to profit from re-irradiation [16,17,51]. We evaluated our cohort based on two established prognostic scores: In our cohort, the DKTK-ROG scoring system shows a better alignment of predicted outcome with survival than the RRRS, but both scores underestimated the mean survival of patients (with a mean underestimation of only 0.5 months for DKTK-ROG). Predictions were especially exceeded by patients with combined chemotherapy, but patients with no chemotherapy had poorer-than-expected survival. This underlines the benefit of alkylating chemotherapy in combination with radiotherapy, but again, limitations might arise for patients unable to undergo chemotherapy and careful screening is mandatory. Similar to both scores and the results from RTOG 1205, patients with initial good ECOG (<2) seem to benefit most from a second course of treatment (*p* = 0.003 for OS). Future developments might also include body composition as an objectively determinable factor, as it shows reduced inter-observer variability compared to KPS or ECOG, which is frequently used at the moment [52].

The selection of patients may help navigate the conflict of balancing aggressive treatment with potential survival benefits while preserving QoL. In a highly diverse patient clientele with GBM patients ranging from not impaired at all to severely limited in everyday activities during all stages of disease, QoL is hard to analyze in a standardized way and data regarding the influence on QoL of different treatment modalities therefore remains limited [2]. Similar to previous retrospective publications, this cohort is inherently subject to selection bias, since initial parameters like age and ECOG heavily influence a physician’s decision regarding the fitting treatment when clear guidelines are missing. Additionally, the use of combination chemotherapy was limited to patients with MGMT promoter methylation, which is widely recognized as positive predictive marker due to the increased effectiveness of chemotherapy in glioma therapy [53,54,55] and demanded as an inclusion criterion for the NOA09/CeTeG trial. In our analysis, MGMT status was significantly linked to prolonged survival after initial diagnosis (*p* < 0.001) but was not predictive for increased survival after re-RT (*p* = 0.18). As discussed previously, the outcome for patients who received combination chemotherapy at recurrence was better than monotherapy, suggesting a stronger statistical effect of the therapeutic regime than the methylation status. This further supports the use of TMZ + CCNU as a combination for re-RT in second-line treatment.

Limitations in this analysis arise from the retrospective nature of the study, which might lead to a selection bias (possibly resulting in favorable PFS and OS) since only patients eligible for re-RT were included, yet it emphasizes the value of second-line treatment for patients in adequate clinical condition. Additional limitations of predictive value of prognostic markers (e.g., MGMT) and treatment specifications (e.g., PTV size, re-RT dose) might be due to the sample size and the inherently limited survival in this patient cohort.

The overall promising outcomes with little side effects in our diverse real-world cohort demonstrated in our analysis show that multimodal treatment should be pursued whenever possible and that re-RT should not be limited to small tumors but serves as a relevant treatment strategy for recurrent glioblastoma, especially in combination with concurrent chemotherapy. In line with other large cohort retrospective analyses, this emphasizes the role of interdisciplinarty treatment in this vulnerable patient cohort [56,57]. Further investigation is needed regarding markers for decision-making regarding treatment concepts and the safety of exceeding dose constraints in organs at risk.

## 5. Conclusions

Re-irradiation with a moderate cumulative EQD2 (~40 Gy) appears to be safe and results in good survival outcomes (mean PFS = 9.5 months, mean OS = 11.3 months), even when applied to larger treatment volumes. Patients amenable to undergo re-resection and receiving concurrent systemic therapy with alkylating agents may have better OS, especially when gross total resection can be achieved. We advocate for a multimodal approach to recurrent glioblastoma with maximal safe resection and adjuvant chemoradiation. The combination of TMZ and CCNU for patients with methylated MGMT promoter yields good results and should be further investigated as a treatment option for recurrent glioblastoma. Normo-fractionated RT enables the use of more generous margins and is tolerated well.

## Figures and Tables

**Table 1:** Patient characteristics. Abbreviations: IDH: isocitrate dehydrogenase-1; MGMT: O-6-methylguanine-DNA methyltransferase; TMZ: temozolomide; CCNU: lomustine; GTR: gross total resection; STR: subtotal resection; ECOG: Eastern Cooperative Oncology Group performance status; PFS: progression-free survival; RT: radiotherapy.

Sex	Male	n = 63 (62%)
	Female	n = 38 (38%)
IDH	Wildtype	n = 91 (90%)
	Mutated	n = 10 (10%)
MGMT	Methylated	n = 62 (61%)
	Non-methylated	n = 39 (39%)
Initial systemic treatment	TMZ (EORTC)	n = 86 (85%)
	TMZ + CCNU (CeTeG)	n = 11 (11%)
	Study	n = 4 (4%)
Additional adjuvant treatment	TTFields	n =8 (8%)
Surgical intervention preceding re-RT	GTR	n = 22 (22%)
	STR	n = 40 (39%)
	Biopsy	n = 15 (15%)
	None	n = 24 (24%)
ECOG pre re-RT	≥2	n = 39 (39%)
	<2	n = 62 (61%)
Corticosteroid dose (Dexa) pre-re-RT	None	n = 76 (75%)
	≤4 mg	n = 17 (17%)
	>4 mg	n = 8 (8%)
Age at recurrence	Median	58.0 years
	95%-CI	55.8–60.2
PFS from end of initial RT to initiation of re-RT	Mean	19.4 months
	Median	12 months
	95%-CI	15.9–22.9

**Table 2:** Details of treatment planning for re-RT.

GTV size [cm^3^]	Median	13.4
	Min	0.3
	Max	143.7
	95%-CI	19.6–30.6
PTV size [cm^3^]	Median	88.1
	Min	21.5
	Max	405.7
	95%-CI	96.2–129.2
GTV-to-PTV margin [mm]	Mean	11
	Min	5
	Max	20
	95%-CI	10.2–11.9
Recurrent GTV covered by iPTV of initial RT [%]	Mean	75
	Min	0
	Max	100
	95%-CI	67–84
Classification of recurrence (following Buglione et al. [14])	In-field	n = 58 (57%)
	Marginal	n = 11 (11%)
	Out-field	n = 14 (14%)
	unknown	n = 18 (18%)
Prescription dose	≤40 Gy	n = 81
	>40 Gy	n = 20
Boost applied	Yes	n = 16
	No	n = 85

**Table 3:** Details of systemic therapy and reported treatment toxicity.

Concomitant systemic therapy	TMZ + CCNU	n = 13
	TMZ	n = 34
	CCNU	n = 33
	None	n = 21
Toxicity assessment		
Decreased blood work parameters	Grade 1	n = 70
	Grade 2	n = 6
	Grade 3	n = 11
	Grade 4	n = 4
Increased liver enzymes	Grade 1	n = 37
	Grade 2	n = 6
	Grade 3	n = 3
	Grade 4	n = 0
Dynamic of steroid intake during re-RT (Dexamethasone equivalent)	Increase	n = 32
	Decrease	n = 9
	Stable	n = 2
	None	n = 58

**Table 4:** Patterns of relapse analysis after re-RT for patients with available MRI-imaging of further progression (n = 57).

	TMZ + CCNU	TMZ	CCNU	None	All
MRI with further progression available	46%	53%	70%	45%	100%
Relapse analysis following Buglione et al. [14]:					
- In-field-relapse	50%	55%	43%	70%	49%
- Marginal relapse	50%	45%	43%	30%	46%
- Out-of-field relapse	0%	0%	13%	0%	5%
Mean relapse volume covered by re-RT PTV [%]	80.7	71.8	60	85.9	74.6
PFS after re-RT					
- Mean	12.6	11.8	4.7	7.1	9.7
- 95%-CI	5.7–19.5	3.1–20.4	3.7–5.7	3.1–11.1	5.8–13.6

## Data Availability

Please contact Niklas Benedikt Pepper regarding data availability.

## Appendix A

**Table A1:** Prognostic factors of patients subcategorized by administered concurrent systemic therapy.

	TMZ + CCNU (n = 13)	TMZ (n = 34)	CCNU (n = 33)	None (n = 21)
Sex				
- Male	64%	50%	76%	60%
- Female	36%	50%	24%	40%
Initial systemic therapy				
- CeTeG	14%	20%	0%	10%
- TMZ (EORTC)	86%	71%	100%	85%
- Trial	0%	9%	0%	5%
Mean PFS initial RT to re-RT [months]	32.0	26.3	11.1	12.8
Age ≥ 50 years	71%	88%	73%	85%
ECOG ≥ 2	36%	41%	27%	55%
MGMT				
- Methylated	100%	88%	30%	50%
- Unmethylated	0%	12%	70%	50%
IDH				
- Mutated	21%	6%	6%	15%
- Wildtype	79%	94%	94%	85%
PTV size > 47 mL	71%	79%	76%	80%
Boost applied	28%	18%	9%	5%
GTV-PTV margins				
≤5 mm	14%	9%	6%	5%
5–10 mm	43%	47%	48%	40%
10–15 mm	29%	38%	22%	40%
≥15 mm	14%	6%	24%	15%
No re-resection performed	29%	47%	30%	50%
DKTK-ROG score				
0–1 points	0%	0%	0%	0%
2–3 points	0%	3%	3%	5%
4–5 points	71%	76%	58%	45%
6–7 points	29%	21%	39%	50%
RRRS				
≤−0.2 (“good”)	14%	0%	3%	0%
−0.2–0.5 (“intermediate”)	57%	59%	70%	45%
≥0.5 (“poor”)	29%	41%	27%	55%

**Table A2:** Toxicity assessment for patients based on concurrent chemotherapy (percentage of patients with respective toxicity incidence).

	TMZ + CCNU	TMZ	CCNU	None
Leukopenia				
Grade 1–2	46%	38%	42%	28%
Grade 3–4	0%	0%	9%	14%
Neutropenia				
Grade 1–2	23%	6%	12%	9%
Grade 3–4	0%	0%	9%	9%
Thrombopenia				
Grade 1–2	61%	70%	66%	38%
Grade 3–4	23%	0%	6%	5%
Elevated liver enzymes				
Grade 1–2	53%	44%	33%	24%
Grade 3–4	0%	3%	3%	0%
